# Comparison of safety between self-expanding metal stents as a bridge to surgery and emergency surgery based on pathology: a meta-analysis

**DOI:** 10.1186/s12893-020-00908-3

**Published:** 2020-10-27

**Authors:** Yang Hu, Jiajun Fan, Yifan Xv, Yingjie Hu, Yuan Ding, Zhengjie Jiang, Qingsong Tao

**Affiliations:** Department of General Surgery, Zhongda Hospital, School of Medicine, Southeast University, Nanjing, Jiangsu China

**Keywords:** Self-expanding metal stents, Bridge to surgery, Pathology, Meta-analysis

## Abstract

**Background:**

To explore the long-term oncological safety of using self-expanding metal stents (SEMS) as a bridge to surgery for acute obstructive colorectal cancer by comparing the pathological results of emergency surgery (ES) with elective surgery after the placement of SEMS.

**Methods:**

Studies comparing SEMS as a bridge to surgery with emergency surgery for acute obstructive colorectal cancer were retrieved through the databases of Pubmed, Embase, and Cochrane libraries, and a meta-analysis was conducted based on the pathological results of the two treatments. Risk ratios (OR) or mean differences (MD) with 95% confidence intervals (CI) were calculated for the outcomes under random effects model.

**Results:**

A total of 27 studies were included, including 3 randomized controlled studies, 2 prospective studies, and 22 retrospective studies, with a total of 3737 patients. The presence of perineural invasion (RR = 0.58, 95% CI 0.48, 0.71, P < 0.00001), lymphovascular invasion (RR = 0.68, 95% CI 0.47, 0.99, P = 0.004) and vascular invasion (RR = 0.66, 95% CI 0.45, 0.99, P = 0.04) in SEMS group were significantly higher than those in ES group, and there was no significant difference in lymphatic invasion (RR = 0.92, 95% CI 0.77, 1.09, P = 0.33). The number of lymph nodes harvested in SEMS group was significantly higher than that in ES group (MD = − 3.18, 95% CI − 4.47, − 1.90, P < 0.00001). While no significant difference was found in the number of positive lymph nodes (MD = − 0.11, 95% CI − 0.63, 0.42, P = 0.69) and N stage [N0 (RR = 1.03, 95% CI 0.92, 1.15, P = 0.60), N1 (RR = 0.99, 95% CI 0.87, 1.14, P = 0.91), N2 (RR = 0.94, 95% CI 0.77, 1.15, P = 0.53)].

**Conclusions:**

SEMS implantation in patients with acute malignant obstructive colorectal cancer may lead to an increase in adverse tumor pathological characteristics, and these characteristics are mostly related to the poor prognosis of colorectal cancer. Although the adverse effect of SEMS on long-term survival has not been demonstrated, their adverse effects cannot be ignored. The use of SEMS as the preferred treatment for patients with resectable obstructive colorectal cancer remains to be carefully weighed, especially when patients are young or the surgical risk is not very high.

## Background

Colorectal cancer is the third largest cancer and the fourth most deadly cancer worldwide today, killing more than 900,000 patients annually [1]. Intestinal obstruction occurs in 8–29% of these patients [2–3]. Once intestinal obstruction occurs, progress is rapid and it will quickly endanger the life of patients. The main treatment in the past was to perform emergency surgery (ES) to relieve the intestinal obstruction. However, due to the water and electrolyte balance disorders, acidosis, and infection caused by intestinal obstruction, the general condition of patients is often very poor. At the same time, the edema of abdominal tissue is serious, resulting in a significantly limited visual field and operation space. Under the influence of these factors, the mortality rates and the incidence of postoperative complications are high [3]. In recent years, self-expanding metal stents (SEMS) have been widely used to relieve intestinal obstruction caused by various benign and malignant diseases. In colorectal cancer, it can serve as a bridge leading to radical surgery for resectable tumors, as well as a palliative therapy for advanced, unresectable tumors. Due to the good general condition and adequate bowel preparation of patients after the placement of SEMS, subsequent elective surgery has obvious advantages in short-term outcomes such as length of hospital stay, primary anastomosis rate and complication rate, but there is still controversy about its long-term oncological results [4]. Several studies have observed the differences and changes of histopathological between SEMS as a bridge to surgery and emergency surgery that was associated with the prognosis of colorectal cancer [5-7]. The purpose of this meta-analysis is to explore the oncological safety of SEMS as a bridge to surgery by comparing the pathological characteristics of tumors between the two treatments.

## Methods

### Search strategy

Searches were performed on PubMed, Embase, and Cochrane Library until April 9, 2020. The following search terms were used for retrieval: Colonial cancer, internal construction, self-expandable metal stent. The combination of medical subject headings (MeSH) and text words were used to search, and relevant articles and references were searched to find as many qualified studies as possible. The search strategy on PubMed is available in Additional file 1.

### Inclusion and exclusion criteria

Inclusion criteria: (1) intestinal obstruction due to colorectal cancer; (2) studies compared SEMS as a bridge to surgery with emergency surgery; (3) reported at least one outcome of interest; (4) all patients involved in the study were judged to be capable of radical tumor resection before operation.

Exclusion criteria: (1) SEMS as a palliative treatment; (2) malignant intestinal obstruction caused by non-colorectal cancer; (3) repeat publication or study with the same data only keep the highest quality one.

### Data extraction

According to the above inclusion and exclusion criteria, two reviewers independently evaluated the eligibility of study selection according to the title and abstract. Then the second round of screening was conducted based on the full text and the final decisions were made. If there was any disagreement between the two reviewers, a third reviewer will join and resolve the disagreement. The extracted data included first author, country, publication year, basic data of patients and tumor pathological results. For continuous variables, extracted the data directly if the data reported in the study were mean and standard deviation. Converted the data to mean or standard deviation if media, standard errors, ranges, or 95% confidence intervals were reported. If the data was incomplete, contacted the author by email to get as much information as possible.

### Outcomes

Outcomes of interest included (1) the TNM stage; (2) the pathological characteristics of the tumor, such as PNI, LVI; (3) the lymph node dissection of the patients, such as the number of lymph nodes harvested and the number of positive lymph nodes.

### Quality assessment

For randomized controlled trials, bias was assessed using the Jadad scale, with a total score of 5 and 3–5 scores for high-quality studies. For prospective and retrospective studies, the Newcastle–Ottawa scale (NOS) was used for evaluation. The total NOS scores were 9, and the scores greater than 6 were considered to be of high quality.

### Statistical analysis

The data were analyzed using RevMan software (Cochrane Review Manager, Version 5.3). For dichotomous variables, risk ratio (RR) and 95% confidence intervals (95% CI) were used. For continuous variables, mean difference (MD) and 95% CI were used. Considering the inherent heterogeneity of the study, such as different selection criteria for SEMS and ES among hospitals, differences in surgical procedures, and so on, we decided to use random effects model only. Heterogeneity of the study was assessed by using the index of I^2^, I^2^ > 50% considered the heterogeneity to be high. If enough studies were included, funnel plots were used to assess publication bias. P < 0.05 was considered statistically significant.

## Results

### Studies selected

**Fig. 1:**
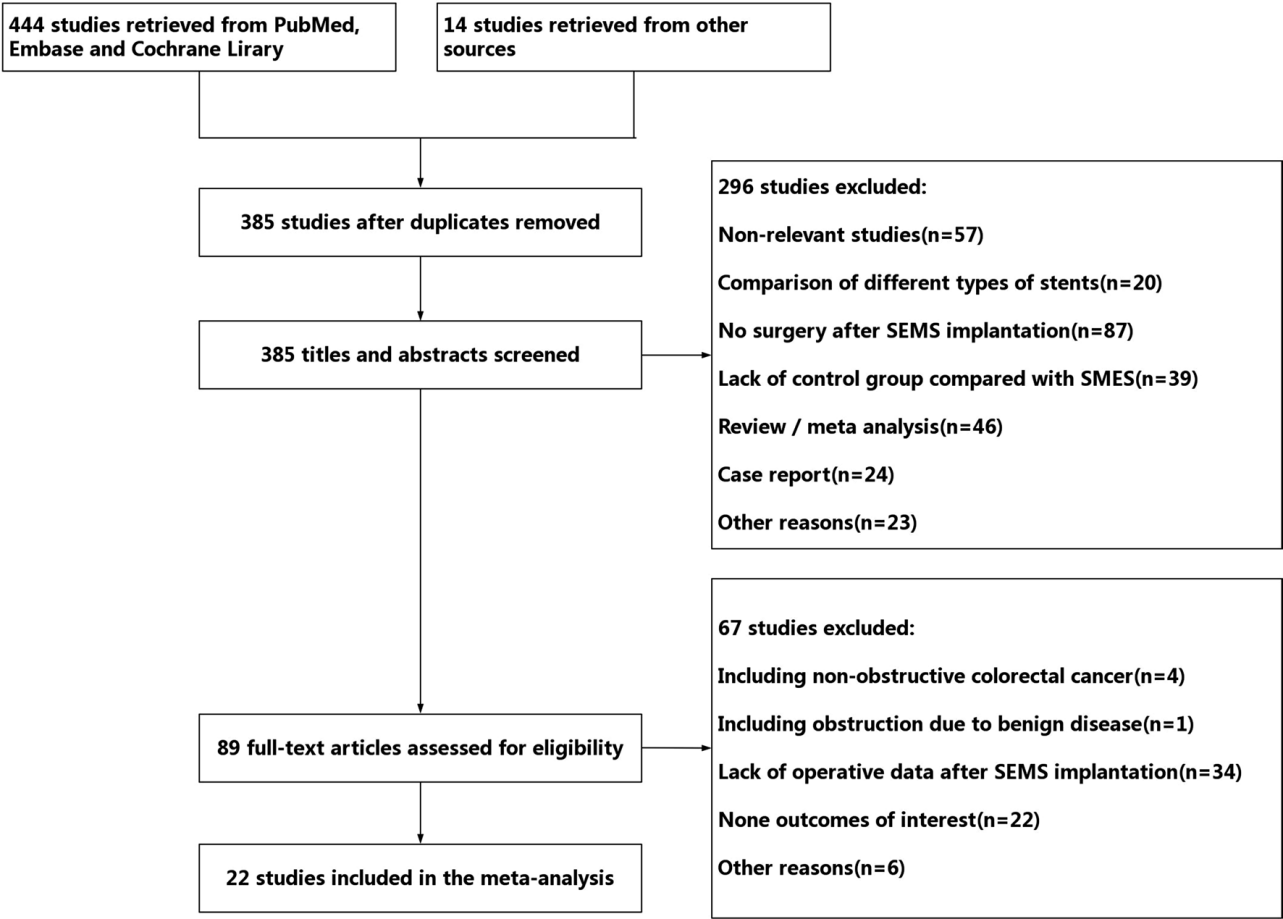
Flowchart for literature inclusion

A total of 458 studies were retrieved according to the search strategy, including 191 studies from Pubmed, 236 studies from Embase, 17 studies from Cochrane Library and, 13 studies from other sources. After screening, 27 studies were included in the final meta-analysis. The studies screening process is shown in Fig. 1.

### Basic characteristics and quality

The basic characteristics and quality evaluation of the included studies are shown in Table 1. A total of 22 studies were included in this meta-analysis, including 3 randomized controlled trials [8–10], 1 prospective study [11], and 18 retrospective studies [12–29]. A total of 1582 patients used SEMS as a bridge to surgery and 1511 patients underwent emergency surgery. All non-randomized controlled trials had NOS scores greater than 6. The Jadad scores of the 3 randomized controlled trials were all greater than 3. It can be considered that all included studies were of high quality.

**Table 1:** Basic characteristics and quality evaluation of included studies

First author	Years	Country	Study type	Obstruction site	Samples SEMS vs ES	Age, mean (range or SD) SEMS vs ES	Gender, M/F SEMS vs ES	NOS/Jadad
Alcantara	2011	Spain	RCT	Left colon	15 vs 13	71.9 (8.96) vs 71.15 (9)	5/10 vs 7/6	3
Amelung	2016	The Netherlands	RS	Left colon	51 vs 37	71.8 (13.1) vs 66.63 (13.2)	25/26 vs 14/23	7
Amelung	2019	The Netherlands	RS	Left colon	222 vs 444	72 (64–80) vs 73 (63–79)	124/98 vs 253/191	8
Arezzo	2017	Italy	RCT	Left colorectal	56 vs 59	72 (43–90) vs 71 (44–94)	28/28 vs 32/27	3
Chen	2019	China	RS	Colorectal	38 vs 90	63.21 (13.55) vs 61.58 (14.84)	23/15 vs 59/31	7
Gorissen	2013	UK	PS	Left colon	62 vs 43	70.6 (28–95) vs 72.0 (36–96)	36/26 vs 20/23	8
Haraguchi	2016	Japan	RS	Colon	22 vs 22	67 (11.0) vs 68 (10.0)	12/10 vs 15/7	6
Ho	2017	China	RS	Left colon	62 vs 40	70.2 (11.7) vs 70.9 (11.5)	49/13 vs 30/10	6
Ji	2017	South Korean	RS	Right colon	14 vs 25	61.5 (14.4) vs 66.9 (12.4)	4/10 vs 11/14	6
Kang	2018	South Korean	RS	Left colorectal	226 vs 109	64.4 (12.8) vs 64.1 (14.8)	141/85 vs 70/39	8
Kavanagh	2013	Ireland	RS	Colon	23 vs 26	69.9 (46–91) vs 69.7 (49–89)	13/10 vs 16/10	7
Kim	2013	South Korean	RS	Left colon	25 vs 70	61.6 (46–80) vs 61.7 (23–90)	15/10 vs 47/23	6
Kim	2015	South Korean	RS	Colorectal	27 vs 29	64.6 (57.8–71.5) vs 70.7 (65.8–75.6)	18/9 vs 16/13	7
Kim	2017	South Korean	RS	Left colon	158 vs 56	63.9 (12.5) vs 64.5 (13.5)	79/79 vs 30/26	6
Kwak	2016	South Korean	RS	Left colorectal	42 VS 42	Not available	28/14 vs 26/16	8
Oistamo	2016	Sweden	RS	Left colon	20 vs 80	Not available	Not available	7
Park	2018	South Korean	RS	Left colorectal	94 vs 17	64.0 (12.1) vs 69.0 (11.5)	52/42 vs 9/8	6
Rodrigues	2019	Portugal	RS	Left colorectal	48 vs 46	67 (58–76) vs 75 (60–83)	25/23 vs 25/21	6
Sabbagh	2013	France	RS	Left colon	48 vs 39	69.73 (13.31) vs 74.89 (13.61)	29/19 vs 20/19	6
Sloothaak	2014	The Netherlands	RCT	Colon	26 vs 32	67 (60–67) vs 70 (61–79)	12/14 vs 18/14	3
Veld	2019	The Netherlands	RS	Left colon	121 vs 121	70.1 (12.1) vs 69.8 (11.0)	73/48 vs 72/29	8
Yang	2019	South Korean	RS	Colon	182 vs 71	65.2 (12.4) vs 63.9 (14.9)	107/75 vs 42/29	6

NOS for PS and RS, Jadad for RCT

*RS* retrospective studies, *RCT* randomized controlled trials, *PS* prospective studies, *SEMS *self-expanding metal stents, *ES* emergency surgery

### Meta-analysis of pathological results

#### PNI (perineural invasion) (Fig. 2)

**Fig. 2:**
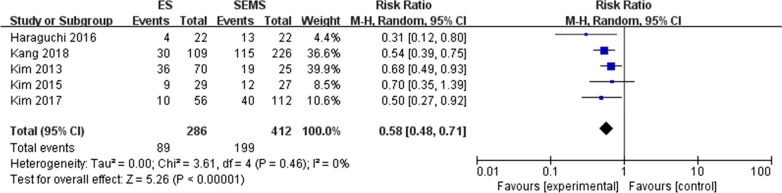
Forest plot of PNI (+) between the SEMS group and the ES group

Five [15, 18, 20–22] of the 22 included studies reported the presence of perineural invasion in pathological specimens. The mean rate of PNI positive in the SEMS group was 48.3% and 31.1% in the ES group, and the difference was statistically significant (RR = 0.58, 95% CI 0.48, 0.71, P < 0.00001).The results of the heterogeneity test showed no significant difference between the two groups (P = 0.46, I^2^ = 0%).

#### LVI (lymphovascular invasion) (Fig. 3)

**Fig. 3:**
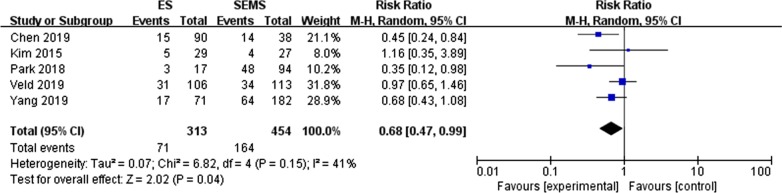
Forest plot of the positive rate of LVI between the SEMS group and the ES group

Five retrospective studies [14, 22, 25, 28–29] reported the occurrence of lymphovascular invasion in pathological specimens of colorectal cancer. Veld [28] subdivided LVI into LI and VI and reported on them separately. The test for overall effect showed that RR was 0.68 (95% CI 0.47, 0.99, P = 0.004) suggested that the rate of LVI positive in the SEMS group was significantly higher than that in the ES group (36.1% versus 22.7%). There was no significant difference between the two groups (P = 0.15, I^2^ = 41%).

#### VI (vascular invasion) (Fig. 4)

**Fig. 4:**
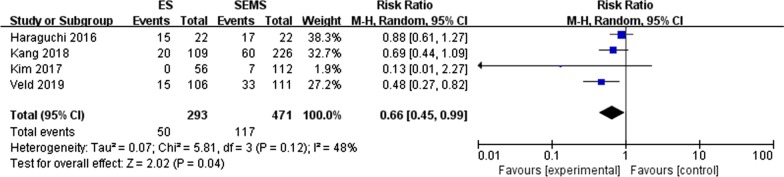
Forest plot of the positive rate of VI between the SEMS group and the ES group

A total of four studies [15, 18, 21, 28] reported the incidence of VI in the SEMS group and the ES group. The results showed that the incidence of VI was 24.8% and 17.1% in the SMES and ES groups, respectively, and the difference between the two groups was statistically significant (RR = 0.66, 95% CI 0.45,0.99, P = 0.04). No heterogeneity was found (P = 0.12, I^2^ = 48%).

#### LI (lymphatic invasion) (Fig. 5)

**Fig. 5:**
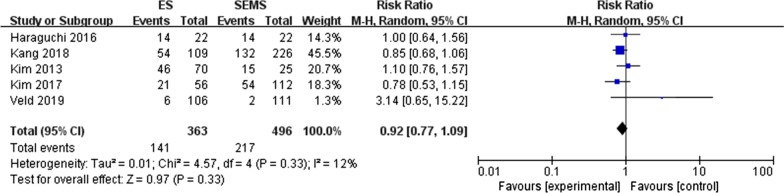
Forest plot for the positive rate of LI of SMES versus ES

Five studies [15, 18, 20–21, 28] reported the invasion of lymphatic vessels by tumor cells. The incidence of LI between the two groups was 38.8% and 43.8%, respectively, with no significant difference exist (RR = 0.92, 95% CI 0.77, 1.09, P = 0.33). Heterogeneity between these studies was low (P = 0.33, I^2^ = 12%).

#### Lymph nodes harvested (Fig. 6)

**Fig. 6:**
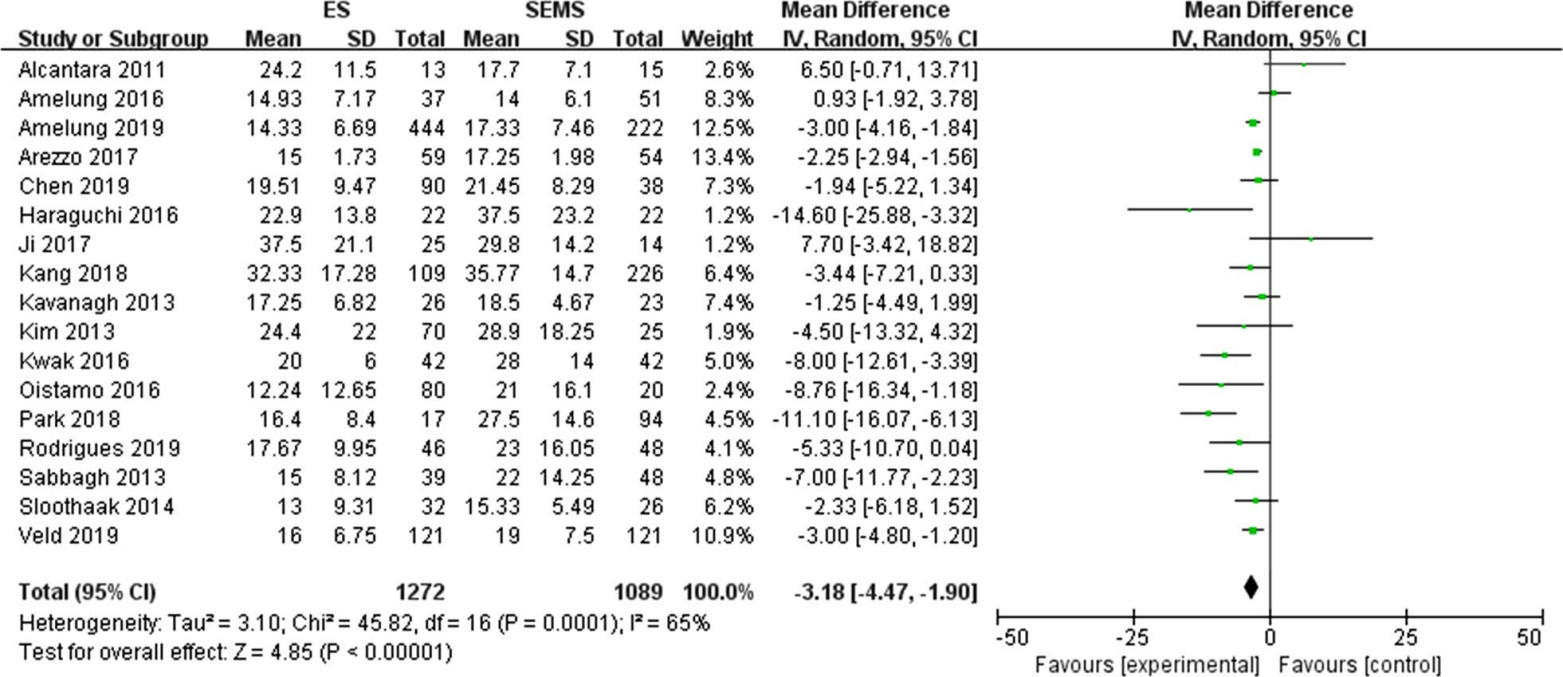
Forest plot of the number of lymph nodes harvested for the two treatments

A total of seventeen [8–10, 12–15, 17–20, 23–28] studies reported the number of lymph nodes dissection. The results showed that elective surgery after the placement of SEMS harvested significantly more lymph nodes than emergency surgery (MD = − 3.18, 95% CI − 4.47, − 1.90, P < 0.00001) with significant heterogeneity among these studies (P = 0.0001, I^2^ = 65%).

#### Positive lymph nodes (Fig. 7)

**Fig. 7:**
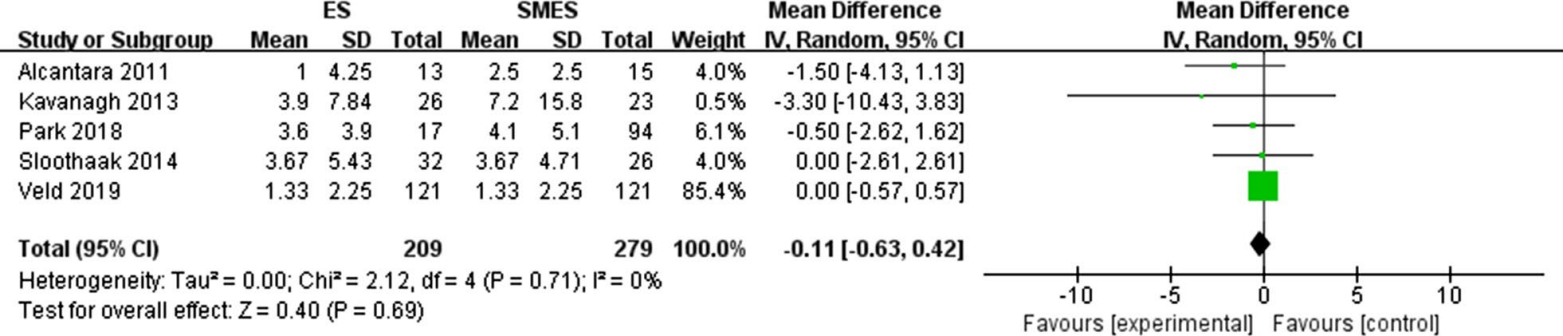
Forest plot of the number of positive lymph nodes between the two groups

Five studies [9–10, 19, 25, 28] reported the number of positive lymph nodes. There was no significant difference between the two groups (MD = − 0.11, 95% CI − -0.63, 0.42, P = 0.69), and no heterogeneity was observed (P = 0.71, I^2^ = 0%).

#### N stage (Fig. 8)

**Fig. 8:**
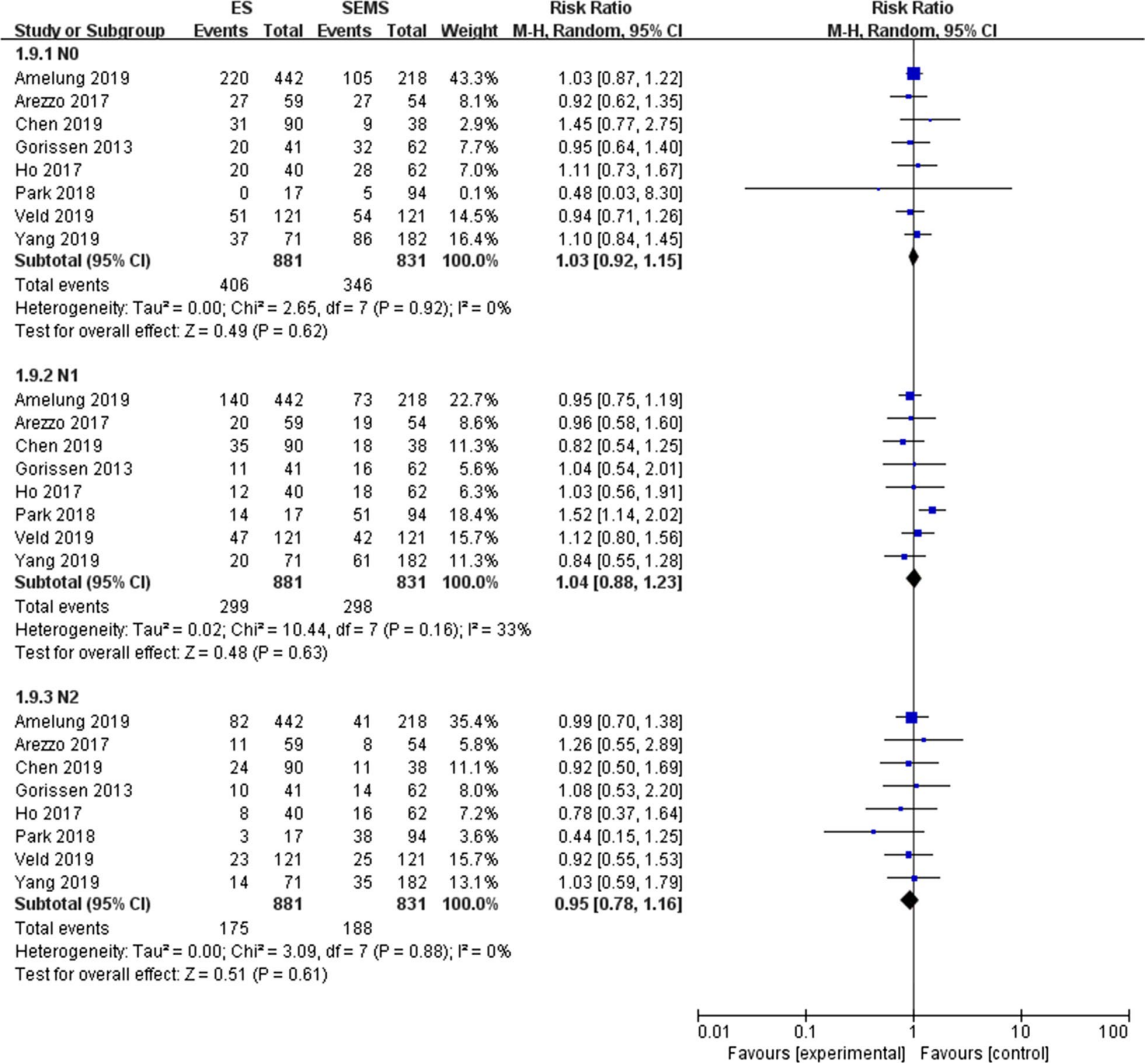
Meta-analysis of the N stage of the two groups

A total of eight studies [8, 11–12, 14, 16, 25, 28–29] reported the N stage of TNM stage in detail. In this meta-analysis N0, N1 and N2 were analyzed respectively, and the results showed that there was no significant difference between the SEMS group and the ES group in the N stage. N0 (RR = 1.03, 95% CI 0.92, 1.15, P = 0.60 and P = 0.92, I^2^ = 0% for heterogeneity), N1 (RR = 0.99, 95% CI 0.87, 1.14, P = 0.91 and P = 0.16, I^2^ = 33% for heterogeneity), N2 (RR = 0.94, 95% CI 0.77, 1.15, P = 0.53 and P = 0.83, I^2^ = 0% for heterogeneity).

### Publication bias and sensitivity analysis

**Fig. 9:**
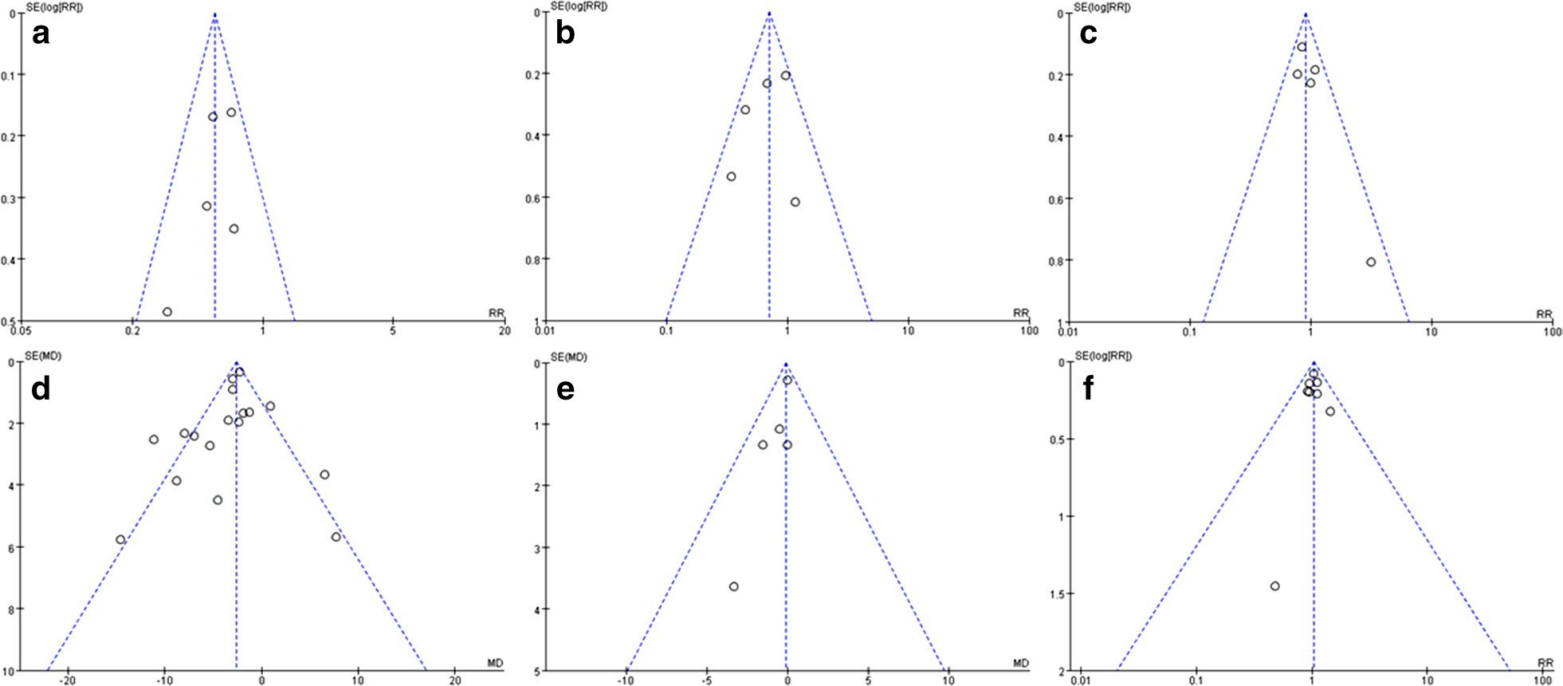
Funnel plots of outcomes **a** PNI, **b** LVI, **c** LI, **d** lymph nodes harvested, **e** positive lymph nodes, **f** N stage

The funnel plots of outcomes with more than 5 studies included are shown in Fig. 9. The results showed that the data were not distributed symmetrically, suggesting the possibility of publication bias. After excluding individual studies one by one, the effect values of the remaining studies and the original effect values did not change significantly, suggesting that the results are relatively stable.

## Discussion

For resectable colorectal cancer, when acute intestinal obstruction occurs, there are currently three main treatment: (1) emergency surgical resection of the tumor, and stoma should be decided according to the patient's condition; (2) elective surgical resection of the tumor after emergency stoma to relieve the intestinal obstruction; (3) elective surgical resection of the tumor after the placement of SEMS. Once intestinal obstruction occurs, there is a high risk of emergency surgery due to various factors such as water-electrolyte balance disorder, acid–base balance disorder, and bacterial translocation, and the mortality rate can be up to 15% [30]. Since the first reported use of SEMS in 1991, SEMS as a bridge of surgery has been widely developed because of its good short-term results, but its long-term oncology results are worrying [4]. Several studies [31–34] have found that SEMS implantation can cause tumor cells to release into the circulatory system, but Ishibashi [31] believes that these tumor cells are not cancer stem-like cells, which can be quickly removed by the body, so they will not cause distant metastasis of the tumor. Some scholars [5–6] also observed many adverse histopathological changes after SEMS implantation, including tumor ulceration, perineural invasion, and lymph node metastasis. However, Matsuda [35] found that the increase of tumor p27kip1 expression level and the decrease of Ki-67 expression level after SEMS insertion, suggesting that the increase of mechanical pressure caused by SEMS may inhibit the proliferation of tumor. In the study of cancer recurrence and disease-free survival rate, etc., the results were also contradictory. Most scholars [20, 36–37] have not observed the difference between the SEMS group and the ES group, while Gorissen [11] believed that SEMS can increase the local recurrence rate of cancer. Sabbagh [27] found the SEMS group was inferior to the ES group in long-term outcomes such as overall survival and cancer-specific mortality apparently. In this meta-analysis, we compared the pathological characteristics of neoplasm between the two treatments to explore the potential oncological results of SEMS as a bridge to surgery.

Perineural invasion is a special pathological feature of many malignant tumors, and the current mainstream view is that PNI refers to the discovery of tumor cells in any of the three layers of nerve structure [38–39]. In colorectal cancer, many scholars believe that PNI positive is an independent prognostic factor for poor prognosis, such as less survival time, shorter recurrence time, and increased local recurrence rate [40–44]. Liebig's [45] research showed that the 5-year disease-free survival rate of PNI negative patients was 4 times higher than that of PNI positive patients, and a previous meta-analysis [46] showed that PNI positive patients with stage II colorectal cancer had a similar postoperative survival to stage III patients. Nozawa [42] found that the increase of mechanical pressure may lead to the development of PNI, and that the SEMS can relieve the intestinal obstruction by expanding the part of the intestine that contains the tumor, no doubt increasing the pressure in that part of the intestine. Many studies have observed a higher PNI positive rate in patients with SEMS [6–7]. The results of this meta-analysis also showed that the PNI positive rate in the SEMS group was significantly higher than that in the ES group. At present, there is no sufficient explanation for the higher PNI positive rate after SEMS implantation, and the increase of mechanical pressure may be one reason. Theoretically, as a recognized independent prognostic factor for colorectal cancer, a higher PNI positive rate would result in a worse prognosis. However, no significant difference was found between the two groups in most studies, which may be due to the following reasons. Firstly, the proportion of postoperative adjuvant chemotherapy is greatly increased due to the acute intestinal obstruction. Previous studies suggested that PNI positive patients could achieve similar survival outcomes as PNI negative patients through adjuvant chemotherapy [43, 46]. Perhaps benefiting from adjuvant chemotherapy, the SEMS group and the ES group achieved similar long-term oncology outcomes. Secondly, the time interval between the placement of SEMS and elective surgery is usually between 5 and 10 days [47], SEMS may lead to more PNI, but the time interval is too short, and the tumor has been radical resected before it can be converted into the effect on long-term oncology results. Besides, since these invaded cells may not be cancer stem cells, have a low malignant potential and maybe quickly recognized and cleared by the body, and therefore do not affect the prognosis of patients. Finally, previous studies have found that mechanical pressure can inhibit the proliferation of tumor cells, while similar phenomena have been observed after SMES implantation [35], and patients may benefit from this to a certain extent.

Lymphovascular invasion refers to the structure of lymphatic or blood vessels invaded by tumor cells, so it can be divided into lymphatic invasion and vascular invasion. Due to the high difficulty in accurately distinguishing lymphatic and vascular in pathological specimens, lymphovascular invasion is generally reported uniformly. In colorectal cancer, lymphovascular invasion is considered to be an independent predictor of poor prognosis [48–49]. It is not only associated with higher T stage and poor differentiation, but also a key link of distant metastasis, and an important risk factor for cancer recurrence and shortened survival [50–52]. In this meta-analysis, we observed a higher rate of lymphovascular invasion in the SEMS group. Some studies suggested that lymphatic invasion and vascular invasion may have different effects on tumors. Compared with lymphatic invasion, vascular invasion is more likely to lead to viscera metastasis [53–54]. The survival time of patients with positive vascular invasion is much lower than that of negative patients, and the number of vascular invasion is positively correlated with recurrence [55–56]. In our meta-analysis, 5 studies reported lymphatic invasion and 4 studies reported vascular invasion respectively, and the results showed that no significant difference was found in lymphatic invasion, while the positive rate of vascular invasion in the SEMS group was significantly higher than that in the ES group. In theory, the prognosis of SEMS group with more lymphovascular invasion or vascular invasion should be worse, but perhaps as with PNI, there is no significant difference in the long-term outcomes between the two groups due to adjuvant chemotherapy, short time interval, non-cancer stem cells invaded and other reasons, but considering the potential adverse effects, SEMS should not be considered as the preferred treatment.

Accurate pathological stage is very important for guiding the postoperative treatment of colorectal cancer, and sufficient lymph nodes dissection is one of the decisive factors to judge the stage of cancer. When the number of lymph nodes in the resected surgical specimens is insufficient, the pathological stage of cancer may be misjudged and the patient cannot receive effective follow-up adjuvant therapy. Meanwhile, the lymph nodes that have been invaded may be omitted, which may lead to the recurrence of cancer and other adverse outcomes. The current study suggests that a low number of lymph nodes harvested can lead to poor prognosis of colorectal cancer. In the emergency surgery for obstructive colorectal cancer, due to severe intestinal dilatation at the upper end of the obstruction and edema of abdominal tissue, the operative field is usually poor, and the difficulty of tissue separation and exposure increases. The placement of SEMS can effectively restore the intestinal patency and make the abdominal tissue edema subside, thus providing a good surgical field of vision. Moreover, the general situation of patients after stent implantation improved significantly, which made the laparoscopy rate in the SEMS group much higher than that in the ES group. The operative field of vision under laparoscopy was broader, which can more clearly show the anatomical structures that are difficult to expose in open surgery. This may explain that in most studies, the number of harvested lymph nodes in the SEMS group was higher than that in the ES group, and our meta-analysis showed the same results. However, there is no significant difference in the number of positive lymph nodes in the meta-analysis. The current guidelines suggest that the number of lymph nodes to be harvested should be more than 12 [57], and most of the included studies were significantly higher than this requirement. At this time, the difference in the number of lymph nodes harvested between the two groups may not affect the pathological stage of patients. This was also confirmed by the meta-analysis of the N stage. Therefore, although the SEMS group has a significant advantage in the number of lymph nodes harvested, this advantage may be of limited significance.

There is no doubt that SEMS, as a bridge to surgery, is superior to emergency surgery in terms of some short outcomes such as primary anastomosis, complications, and permanent stoma rate [4], but its histopathological performance is significantly inferior to emergency surgery. The complications of SEMS placement also can not be ignored. Perforation, as the most serious complication, will not only cause peritoneal dissemination of tumors, but also be the main cause of early death. According to the literature, its incidence is as high as 7.4%, while the rate of occult perforation is higher [5, 58]. Overall survival (OS) and disease-free survival (DFS) are the most commonly used indicators to evaluate the prognosis of cancer patients. A recent literature review showed that only a small number of studies showed that the placement of SEMS may affect the long-term surgical outcomes, and most studies showed that SEMS did not have a negative impact on the patients’ long-term survival and prognosis [59]. Two recent meta-analyses respectively summarize the data of 2508 patients and 15,224 patients, the results showed no significant difference between SEMS and ES in terms of three-year OS and three-year DFS, or five-year OS and five-year DFS, which was consistent with the results of previous meta-analysis [59–61]. However, given the relatively small proportion of randomized controlled studies (20.8% and 26.7% respectively), and the factors such as stent placement technology and stent type can not be standardized, and due to the possible influence of factors such as chemotherapy and surgical interval mentioned above, the absence of significant differences in long-term surgical outcomes should be interpreted with caution. In summary, there is a risk of using SEMS as the preferred treatment for patients with resectable tumors. The European Society of Gastrointestinal Endoscopy (ESGE) has not recommended the routine use of SEMS as a bridge to elective surgery for left-sided malignant intestinal obstruction, but SEMS can be considered as a bridge for elective surgery in potentially curable patients ASA ≥ III and/or age > 70 years [47].

This meta-analysis compared the pathological characteristics of the SEMS group and the ES group to explore the long-term oncology safety of SEMS as a surgical bridge, but many limitations of this study may affect the interpretation of the results. First, twenty-two of the 27 studies were retrospective (about 81.5%) with inherent limitations, and its’ large proportion required careful interpretation of the results. Furthermore, the three included RCTs also failed to achieve blind allocation between doctors and patients. Secondly, there was high heterogeneity among studies, such as different types of self-expanding metal stents, the proficiency of endoscopists, the experience of the center, different emergency surgical procedures in different hospitals, and different selection criteria for SEMS and emergency surgery, etc. Third, the definition of some pathological features is currently controversial. For example, PNI may be missed or misreported due to the inconsistent definition, and the authenticity of the results may be affected. A similar situation may also exist in LVI.

## Conclusions

After the placement of SEMS, some important pathological features that affect the prognosis of patients, such as perineural invasion, lymphovascular infiltration, etc., increased significantly. Although it may not affect the long-term survival of patients under the influence of multiple factors, the benefits and risks of using SEMS as the preferred treatment for resectable colorectal cancer should be carefully weighed. At present, surgical techniques and instruments have made great progress. When patients are young or the surgical risk is not very high, emergency surgery may make patients obtain better oncological outcomes due to possible adverse effects of SEMS. For patients with an increased risk of postoperative mortality, SEMS as a bridge to elective surgery may achieve greater benefits than emergency surgery.

## Supplementary information

**Additional file 1:** Search strategy on PubMed

## Data Availability

All data generated or analysed during this study are included in this published article.

## Abbreviations

SEMS: Self-expanding metal stents
ES: Emergency surgery
NOS: Newcastle–Ottawa scale
PNI: Perineural invasion
LVI: Lymphovascular invasion
VI: Vascular invasion
LI: Lymphatic invasion
CI: Confidence interval
MD: Mean differences
RR: Risk ratio
