# Supplementary material for: Comparison of safety between self-expanding metal stents as a bridge to surgery and emergency surgery based on pathology: a meta-analysis
Source: BMC Surg. 2020 Oct 27;20:255. doi: 10.1186/s12893-020-00908-3 (PMC7592574; doi:10.1186/s12893-020-00908-3)
Supplement: Supplementary file 1 — Additional file 1: Search strategy on PubMed [file 12893_2020_908_MOESM1_ESM.doc]

**Search [strategy](../../../../D:/Program%20Files%20(x86)/Dict/8.9.2.0/resultui/html/index.html#/javascript:;) on PubMed**

(("Colorectal Neoplasms"[MeSH Terms] OR (((((((((((((("colorectal neoplasm"[Title/Abstract] OR "neoplasm colorectal"[Title/Abstract]) OR "colorectal carcinoma"[Title/Abstract]) OR "carcinoma colorectal"[Title/Abstract]) OR "carcinomas colorectal"[Title/Abstract]) OR "colorectal carcinomas"[Title/Abstract]) OR "colorectal cancer"[Title/Abstract]) OR "cancer colorectal"[Title/Abstract]) OR "cancers colorectal"[Title/Abstract]) OR "colorectal cancers"[Title/Abstract]) OR "colorectal tumors"[Title/Abstract]) OR "colorectal tumor"[Title/Abstract]) OR "tumor colorectal"[Title/Abstract]) OR "tumors colorectal"[Title/Abstract]) OR "neoplasms colorectal"[Title/Abstract])) AND ("Intestinal Obstruction"[MeSH Terms] OR ("intestinal obstructions"[Title/Abstract] OR "obstruction intestinal"[Title/Abstract]))) AND ((("self expandable metallic stent"[Title/Abstract] OR "self expandable metal stent"[Title/Abstract]) OR "self expandable metal stents"[Title/Abstract]) OR "Self Expandable Metallic Stents"[MeSH Terms])
